# Acoustic frequency comb generation on a composite diamond/silicon microcantilever in ambient air

**DOI:** 10.1038/s41378-025-00866-x

**Published:** 2025-01-17

**Authors:** Zhixin Zhao, Yanyan Li, Wangyang Zhang, Wenyao Luo, Duo Liu

**Affiliations:** Institute of Novel Semiconductors, State Key Laboratory of Crystal Materials, Shandong University, 27 South Shanda Road, Shandong, 250100 P. R. China

**Keywords:** Physics, Materials science

## Abstract

Acoustic frequency combs (AFCs) contain equidistant coherent signals with unconventional possibilities on metrology. Previously, implementation of AFCs on mechanical microresonators with large air damping loss is difficult, which restricted their atmospheric applications. In this work, we explore the potentials of a composite diamond/silicon microcantilever for parametric generation of AFCs in ambient air. We discover that the diamond layer provides a viable route to reduce the linewidth of the primary flexural mode, yielding a 7.1-times increase of the quality factor. We develop a parametric driving scheme that enables generation of AFCs through injection locking and sequential nonlinear dynamic transitions involving subharmonic synchronization (Arnold tongue), and chaotic dynamics. Ultimately, we realize AFCs with a frequency range extending 800 kHz in the air. This work advances the understanding of AFCs and provides a viable route towards their applications in ambient air for high precision metrology.

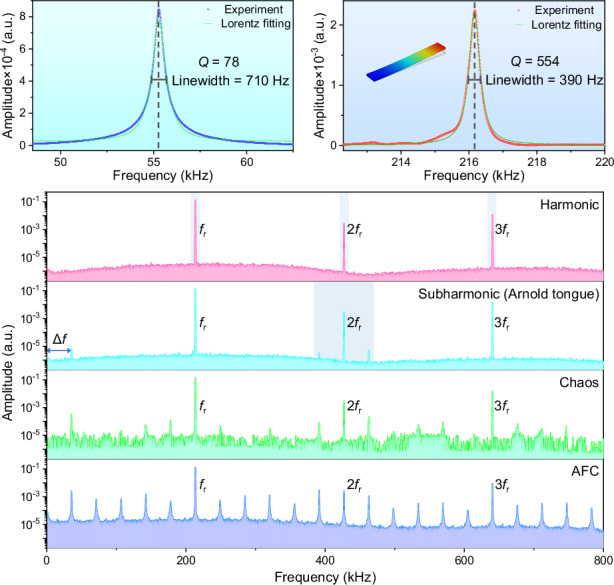

## Introduction

Since its inception in 1990 s, optical frequency combs (OFCs) have been routinely realized in optical system and catalyzed remarkable advance in precise metrology^1,2^, telecommunications^3,4^, molecular fingerprinting^5^, optical atomic clock^6^, astrophysics^7^, and broadband gas sensing^8^. Acoustic frequency combs (AFCs) are mechanical analog to OFCs, which produces acoustic signals at a precise set of frequencies, with potential to be used to measure various physical, chemical and biological quantities in which acoustic waves can propagate.

Up to date, the majority of reported AFCs have been obtained in vacuum environment through intermodal interactions and they often exhibit only a few comb lines^9,10^. This prerequisite brings great inconvenience to real applications of AFCs as many physical sensing and biochemical detection works need to be carried out in viscous gas or fluidic environment. The quality (*Q*) factor, which measures the energy dissipation rate in each cycle of oscillations, is a key parameter that needs to be optimized for development of high-performance microelectromechanical system (MEMS) resonators^11^. The higher the *Q* factor, the longer the time in which energy will remain in the devices, which facilitate intermodal energy transfer. Diamond, with an ultrahigh Young’s modulus (~1100 GPa), a low thermal expansion coefficient (~2.6 × 10^−6^/K) and intrinsic hydrophobic surface^12^, is a potential material candidate to increase *Q* factor in MEMS resonators.

Arnold tongue^13^ is frequently encountered in the study of nonlinear systems, ranging from electrical circuits^14^, neuroscience^15^, coupled resonators^16^, to biological systems^17^. The formation of Arnold tongue is governed by the delicate balance between the natural frequencies of the oscillators and the influence of the coupling strength. It refers to the region in parameter space where synchronization exists between oscillators at specific frequency detuning for the generation of harmonics, subharmonics, and rational harmonics^18–20^.

**Fig. 1:**
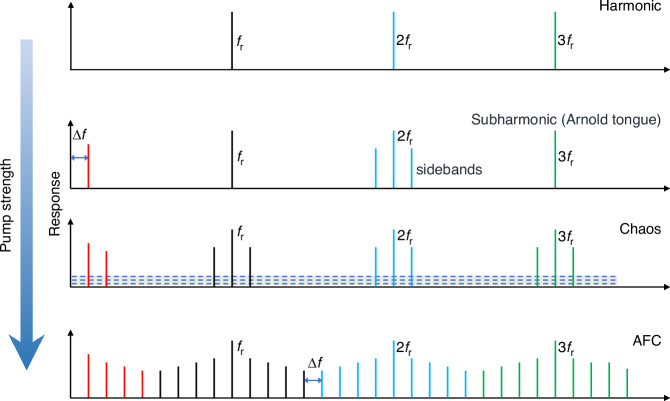
Schematic for the formation process of AFCs. With the increase of pump strength, harmonics, subharmonics (Δf, Arnold tongue), sidebands, chaos and AFCs appear successively

In this Article, we demonstrate the generation of AFCs on a high *Q* composite diamond/silicon microcantilever resonator in ambient air. This is achieved through parametric pumping at the primary flexural mode (*f*_r_) through strength adjustment. The system experienced a complex nonlinear dynamic process in terms of the pumping strength (Fig. 1) through sequentially formed harmonics (2*f*_r_, 3*f*_r_) and synchronization induced subharmonics (Δ*f*, Arnold tongue). The system then undergoes a chaotic conversion with energy spreading over the full spectrum region. Above a threshold, we observe chaos-assisted formation of AFCs through cascaded parametric processes.

## Results

### High *Q* diamond/silicon microcantilever

The diamond/silicon microcantilever used in the study contains a 3.5 μm thick diamond layer deposited on a silicon microcantilever (218 × 32 × 2 μm^3^) using microwave plasma assisted chemical vapor deposition (MPCVD). The details on the growth are included in the “Methods” section. We also systematically examined the surface morphology, spatial variations of the stress distribution, surface chemical composition of the diamond layer by Scanning Electron Microscope (SEM), Raman and X-ray photoemission spectroscopy (XPS) respectively (see Supplementary Note 1). SEM reveals that the diamond/silicon microcantilever surface remains flat and uniform after diamond deposition (Supplementary Fig. 1a). Raman spectroscopy indicates that the stress is minimal and uniformly distributed (Supplementary Fig. 1b, d). XPS shows that the oxygen content on the diamond surface is lower than that of silicon (Supplementary Fig. 1e).

**Fig. 2:**
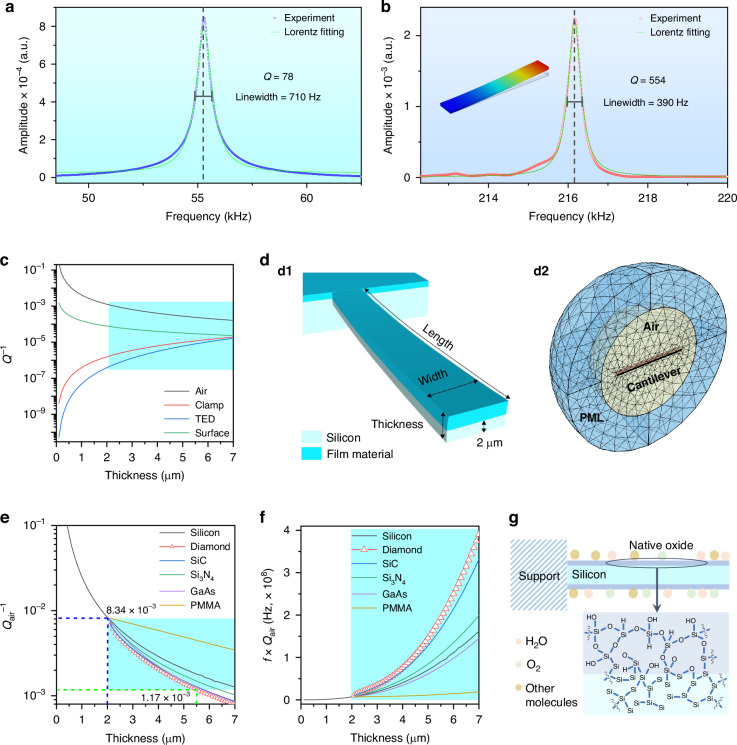
High Q diamond/silicon microcantilever. Amplitude frequency spectra around the primary flexural mode of the silicon microcantilever beam (**a**) and the diamond/silicon microcantilever (**b**). The *Q* factors for the two beams are determined to be 78 and 554, respectively. **c** Variation of the energy dissipation terms in Eq. (1) with respect to the beam thickness. **d** Schematic for calculating the air damping loss of the composite diamond/silicon microcantilever. **d1** Schematic of the composite diamond/silicon microcantilever with size label. **d2** The 3D model (after mesh) used for calculating the air damping loss. **e, f** Calculated ${Q}_{\text{air}}^{-1}$ and *f* × *Q*_air_ for various composite microcantilevers coated with different materials. **g** Schematic of surface loss caused by surface stress caused by oxide layer and adsorbent on silicon

Figure 2a, b shows the frequency responses of the monolithic silicon and the diamond/silicon microcantilevers around the primary flexural modes in the air. The eigenfrequencies *f*_r_ of the primary flexural modes for the two beams are located at 55.29 kHz and 216.16 kHz, respectively. The *Q* factors are determined by Lorentzian fitting to be 78 and 554, respectively, signifying a 7.1-times enhancement. The underlying principle for the observation is discussed as follows.

For a microcantilever operating in the air, the overall *Q* factor *Q*_total_ is determined by^21^:

1 $${\text{Q}}_{\text{total}}^{-1}={\text{Q}}_{\text{air}}^{-1}{+\text{Q}}_{\text{clamp}}^{-1}+{\text{Q}}_{\text{TED}}^{-1}+{\text{Q}}_{\text{surface}}^{-1}$$

where energy loss channels associated with air damping loss ${\,Q}_{\text{air}}^{-1}$, clamp loss ${Q}_{\text{clamp}}^{-1}$, thermoelastic loss${\,Q}_{\text{TED}}^{-1}$, and surface loss ${Q}_{\text{surface}\,}^{-1}$ are considered. We explicitly calculated each *Q* for a monolithic silicon microcantilever (218 × 32 μm^2^) as a function of the beam thicknesses (Fig. 2c). The equations used for the calculation of the *Q* factors of a microcantilever have been well documented^22–25^ and are presented in Supplementary Note 2. As the thickness increases, both ${\,Q}_{\text{air}}^{-1}$ and ${Q}_{\text{surface}\,}^{-1}$ decrease, while ${Q}_{\text{clamp}}^{-1}$ and ${\,Q}_{\text{TED}}^{-1}$ increase. For a thin microcantilever (<5.5 μm), ${\,Q}_{\text{air}}^{-1}$ and ${Q}_{\text{surface}\,}^{-1}$ are orders of magnitude greater than the sum of ${Q}_{\text{clamp}}^{-1}$ and ${\,Q}_{\text{TED}}^{-1}$, such that ${Q}_{\text{air}}^{-1}\gg {Q}_{\text{surface}}^{-1}\gg {\,Q}_{\text{clamp}}^{-1}+{Q}_{\text{TED}}^{-1}$.

We then undertake an assessment of the impact of various coating materials, i.e. silicon, diamond, SiC, Si_3_N_4_, GaAs, and polymethyl methacrylate (PMMA) on ${Q}_{\text{air}}^{-1}$ by using finite element method (FEM) simulation (Fig. 2d). The simulation utilized a thermoviscous acoustic–shell interaction was modeled for fluid -solid coupling. Figure 2e, f reveal a decrease in ${Q}_{\text{air}}^{-1}$ with an increase of the thickness of the coating layer, similar to the trend observed in the monolithic silicon microcantilever (Fig. 2c). In particular, diamond stands out as the optimal material choice for reducing ${Q}_{\text{air}}^{-1}$, signifying a ~ 7-times reduction from 8.34 × 10^−3^ to 1.17 × 10^−3^ for a film thickness of 3.5 μm. This result can be attributed to the exceptionally high Young’s modulus of diamond. SiC is also found to be an excellent material choice for cantilever modification, while soft materials, e.g. PMMA, would reduce ${Q}_{\text{air}}^{-1}$. The diamond/silicon microcantilever also shows a higher ${f}\times {{Q}}_{\text{air}}$ product, indicating a more efficient and stable performance and higher sensitivity^26^.

Surface loss also constitutes a majority of the total energy loss for microcantilever operating in the air. Previous studies revealed that the surface loss arises from intrinsic surface thermoelastic dissipation, chemical adsorbent^27^, extrinsic thermoelastic dissipation related to defective surface layer^28^. Silicon has a naturally developed silica layer that could be formed during the fabrication process of the microcantilevers also involves the usage of silica as sacrificial layer to obtain desired patterns. The silica layer is usually defective, and contains strained Si-O network or dangling bonds^29^ with enhanced energy dissipation during oscillation. Besides, silica is hydrophilic in nature, resulting in surface accumulation of water, oxygen, and polar molecules^30^ (Fig. 2g). The absorption and desorption of these molecules also constitutes a portion of energy dissipation^31^. This adverse effect can be alleviated by application of a diamond protection layer, which is known for its hydrophobic nature (See the Supplementary Note 1 for measured water contact angle) and high Young’s modulus.

### AFCs generation

For a regular silicon microcantilever, nonlinear kinetic responses are usually observed in vacuum due to the prerequisite of high *Q*. We explored the potential of the high *Q* diamond/silicon microcantilever for AFCs generation in the air. To achieve this, the microcantilever was resonantly driven at the vicinity of *f*_r_ by a sinusoidal pumping frequency *f*_pump_ with gradually increased pump voltages *V*_pump_.

**Fig. 3:**
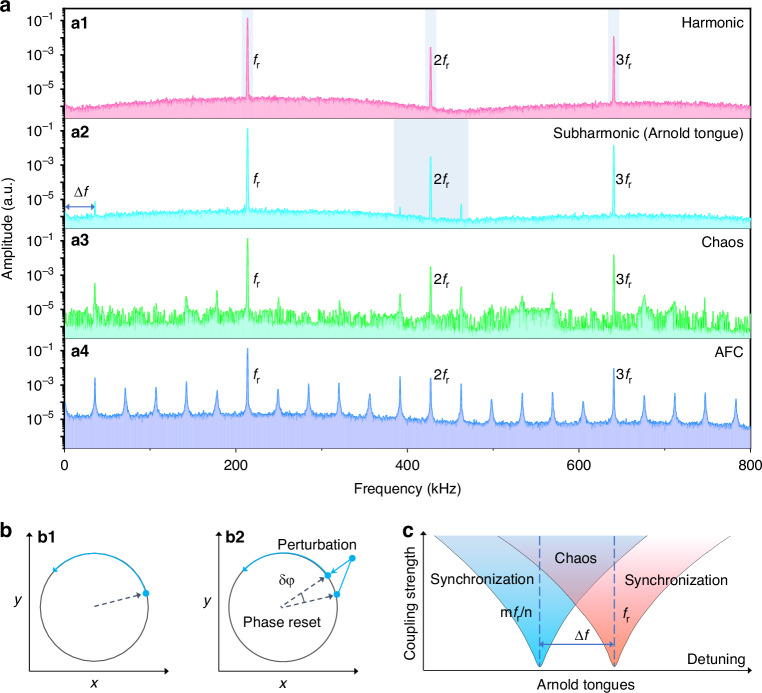
Formation process of AFCs. **a** Measured spectra showing the formation of AFCs by parametric excitation at the primary frequency *f*_r_ with increasing *V*_pump_. **a1** harmonics at 2*f*_r_ and 3*f*_r_. **a2** subharmonic synchronization (Arnold tongue). **a3** Chaos. **a4** AFCs formed by FWM. **b** The limit cycle in phase space. **b1** A limit cycle in phase space showing a closed trajectory for a free running resonator. **b2** Resetting the phase δφ in a limit cycle by periodic perturbations. **c** The periodic perturbations induce the Arnold tongues featuring rational subharmonics

Figure 3a shows the spectral evolution of the amplitude dependent frequency responses of the microcantilever. The dynamics contains four distinct stages. First of all, 2nd harmonic and 3rd harmonics (noise background ~1 × 10^−6^) at 2*f*_r_ and 3*f*_r_ were observed when *V*_pump_ reached a threshold voltage, i.e. 650 mV (Fig. 3a1). The 3rd harmonic arises from Duffing nonlinearity, while the 2nd harmonic may be related to geometric nonlinearity caused by symmetry breaking. On increasing *V*_pump_ to 660 mV rational synchronization with a subharmonic formed at Δ*f* = *f*_r_/m (m = 6, in this case) was observed, i.e. the Arnold tongue (Fig. 3a2). Simultaneously, two sidebands at 2*f*_r_ ± Δ*f* appeared around the 2nd harmonic due to sum and difference frequency generation (SFG and DFG). Further increasing *V*_pump_ to 660 mV creates a turbulent state that contains a chaos background (3 × 10^−6^) with enhanced noises and multiple sidebands around the harmonics through four-wave mixing (FWM)^32^, suggesting energy spreading over the whole spectral region (Fig. 3a3). The chaotic outputs, which are intrinsically unpredictable, had been proposed to function as “chaotic encryption”^33^. Finally, transition from chaos to frequency combs occurs and the frequencies of the comb lines meet *f* = *f*_r_ + nΔ*f*, resulting in an AFC (noise background ~ 3 × 10^−5^) spanning 800 kHz with a frequency interval Δ*f* equal to a rational multiple (1/6) of *f*_pump_ (Fig. 3a4).

Subharmonic synchronizations are not always manifested and could be observed when the oscillator is phase locked by external pump^34^. In general, mode locking appears in region the frequencies locks exactly into a rational ratio *m*/*n*, when a harmonic m*f*_r_ approaches another harmonic n*f*_r_, resulting in the formation of parameter region called “Arnold tongue”. The theoretical framework to understand Arnold tongues depends on the phases of the vibrational mode. A stationary self-sustained oscillation can be described by the limit cycle in the phase space (Fig. 3b1). An external perturbation induces a phase difference (Fig. 3b2) is an integer rational of the primary frequency of the system, which could be described by the Kuramoto model for a synchronous system^35^. Arnold tongues manifest when a perturbation effectively compensates for the detuning between the pump signal and the intrinsic frequency of the oscillator. Once a critical coupling strength is reached, the tongues could overlap with each other, leading to a state with multiple stable solutions (Fig. 3c). Chaotic behavior arises because of resonant interaction of different modes in the overlapped region such that orbit jumps could occur in an erratic way that promotes the noise floor in the system^36^^.^ Further increasing the pumping strength promotes chaos assisted synchronization^37^ to form AFCs through cascaded FWM.

Synchronization and AFCs could occur in a self-sustained resonator with forced vibration, and the dynamics of the system is given in Supplementary Information Eq. (S5, S6). The Eq. (S5, S6) can be numerically evaluated to obtain AFCs. It is this phase difference caused by the external periodic excitation that leads to the periodic modulation of the amplitude, thereby generating AFCs.

### Frequency detuning

The presence of Arnold tongues depends on the pump voltage and the frequency detuning. When detuning is minimal, a minor external perturbation can suffice to synchronize an oscillator. Conversely, when detuning is pronounced, a strong signal and its corresponding reaction are essential for the emergence of Arnold tongues.

**Fig. 4:**
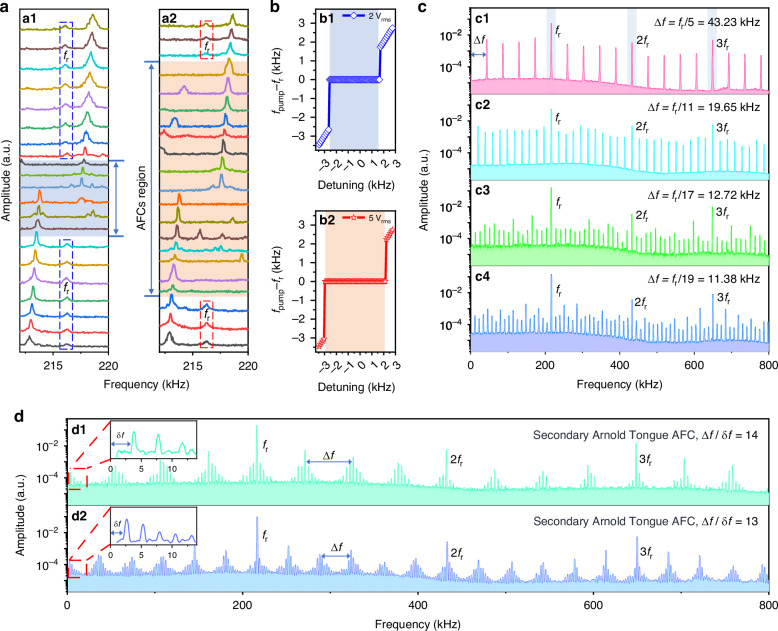
Frequency detuning. **a** Measured spectra showing the primary frequency *f*_r_ can be locked by *f*_pump_ though modulating *f*_pump_ at a pump voltage of *V*_pump_ = 2 V_rms_ and 5 V_rms_. **b** Frequency mismatch (*f*_pump_ – *f*_r_) versus detuning plot for a certain fixed *V*_pump_. Synchronization is exhibited in the specific detuning range wherein *f*_pump_ – *f*_r_ = 0. **c** Measured spectra by frequency detuning at a fixed *V*_pump_ = 2 V_rms_. AFCs with a frequency interval Δ*f* = *f*_r_/5 (**c1**), *f*_r_/11 (**c2**), *f*_r_/17 (**c3**) and *f*_r_/19 (**c4**). **d** Secondary Arnold tongue AFCs

We then explored the effects of frequency detuning on the system’s responses under a constant *V*_pump_ of 2 V_rms_ and 5 V_rms_. As shown in Fig. 4a1, a2, the primary frequency *f*_r_ can be locked by *f*_pump_ in the range of 213.6 kHz to 217.8 kHz and 213.2 kHz to 218.3 kHz, respectively, resulting in synchronization. Fig. 4b1, b2 reveal the synchronization range of 4.2 kHz and 5.1 kHz, respectively. The observed injection locking range align well with the prevailing principles of injection locking. For a self-sustained oscillator, the maximum locking range^38^ is determined by the amplitude ratio of the injection-locked oscillation *V*_pump_ and the *Q* factor (${V}_{\text{pump}}/Q$). For *V*_pump_ = 5 V_rms_, this locking range is 13.1-times the line width of the primary mode. We also found injection locking is crucial for the formation of the AFCs, which can only be formed in the frequency region where the primary frequency *f*_r_ is locked by *f*_pump_. Our results reveal that the locking range, determined by a combination of the pumping frequency, the mode frequency, and the *Q* factor, serves as the boundary of the Arnold tongue and also determines the existence region of AFCs^37,39^.

Figure 4c shows the spectral evolutions of AFCs by detuning *f*_pump_ for *V*_pump_ = 2 V_rms_. We observed that the frequency intervals Δ*f* can be easily tuned by changing the detuning. Figure 4c1, c2, c3, and c4 shows AFCs with Δ*f* equals to 1/5, 1/11, 1/17 and 1/19 of *f*_pump_, respectively. Additionally, under certain conditions, it is possible to create secondary subharmonics with a frequency interval δ*f* with a near commensurate ratio that obey the relationship Δ*f*/δ*f* = 13.995 and 12.990 (Fig. 4d1, d2).

### Stability of AFCs

**Fig. 5:**
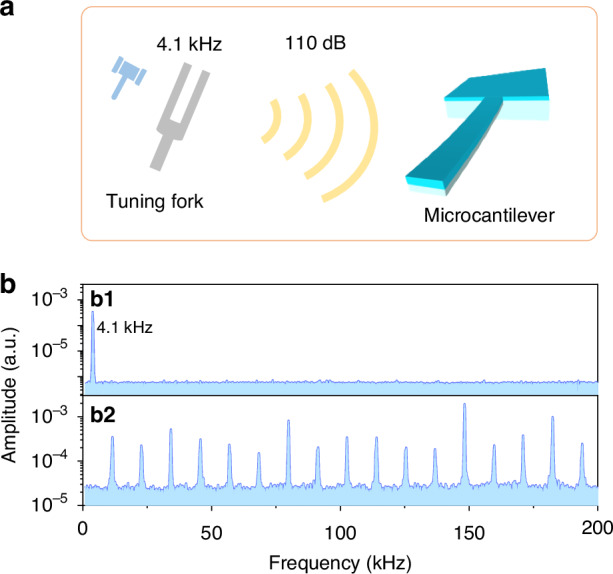
Anti-interference performance of AFCs. **a** Schematic of striking a tuning fork (4.1 kHz) to produce a 110 dB sound. **b** Measured vibrational spectra without (**b1**) and with (**b2**) AFCs when the tuning fork is struck

Unlike our recent work on silicon cantilever in vacuum environment^40^, this present work could significantly expand the applications of AFCs in ambient air. We investigated the stability of the AFCs when subjected to perturbation from an intense sound wave produced by a tuning fork with continuous tapping at 4.1 kHz (Fig. 5a). The sound power level was measured to be 110 dB using a power meter. To assess the frequency stability of the microcantilever, we measured the frequency response of the diamond/silicon microcantilever before and after the formation of AFCs under the influence of the sound waves. Before the formation of AFCs, the microcantilever responded at 4.1 kHz due to interaction with the sound waves from the tuning fork (Fig. 5b1). Once AFCs formed, the microcantilever became immune to the sound waves, without any response at 4.1 kHz, and the AFCs teeth remains to be stable over the whole frequency range without any detectable frequency shift (Fig. 5b2). The high stability of the AFCs against sound perturbation could be attributed mode locking. As all modes are phase-locked to each other to form a stable vibrational pattern. This coherence between the modes enhances stability because any perturbation is countered by the collective behavior of all the modes working to maintain phase coherence. This observation proves excellent stability of the AFCs against acoustic disturbance, with potentials for applications that demand high frequency stability in high-precision acoustic measurements.

## Discussion

In summary, we report the fabrication of a composite diamond/silicon microcantilevers using MPCVD technique. Specifically, diamond has been identified as an optimal material to notably reduce air damping and surface loss with a largely enhanced *Q* factor (~7.1-times of the bare silicon microcantilever). The system is highly controllable and exhibits abundant nonlinear dynamic behaviors, which include harmonic responses, subharmonic synchronization, wave mixing, chaotic vibration, and ultimately the development of AFCs. In specific, the teeth of the AFCs can be easily adjusted by parametric detuning of the pump frequency. The results are discussed based on injection locking, Arnold tongue, and chaotic orbit jump due to resonant interactions between different modes within the overlapping frequency. We hope that this present work would advance the understanding of AFCs and provides a viable route towards its applications in ambient air for high precision metrology.

## Materials and methods

### Sample fabrication

First, commercial detonation nanodiamonds (NDs) (50 nm in diameter) was annealed in oxygen at 400 °C for 4 h to reduce graphite and other carbon species. The NDs were then ultrasonically dispersed in pure water (1.5 mg.mL^−1^), followed by centrifugation for 6 h at 1000 rpm. The supernatant NDs colloidal suspension (negatively charged) was collected for seeding application. the silicon microcantilever was treated with cationic methacrylatoethyl trimethyl ammonium chloride (DMC, 10 vot%, Mw: 207.7) solution for 10 min, which allows electrostatic attachment of anionic NDs. The microcantilever were then placed in a MPCVD apparatus (UP-206, Uniplasma, China) for diamond growth. During growth, the microwave power was fixed at 3300 W, and the H_2_ and CH_4_ flow rates were maintained at 500 and 20 sccm under a chamber pressure of 120 torr, respectively. The growth lasted approximately 65 min and the film thickness was estimated to be 3–4 μm. After growth, the sample was etched in H_2_ plasma for 50 min to reduce the surface carbon species, and the sample was then allowed to cool slowly down to room temperature in H_2_ atmosphere.

### Experimental setup

To investigate the frequency response and dynamic behaviors, the microcantilever were glued on a lead zirconate titanate (PZT) transducer with a d_33_ value of approximately 250 pm.V^−1^. For cantilever excitation, sinusoidal (AC) pump signals were applied with a function generator (33220 A, Agilent, USA) and the frequency response spectra were recorded by using a Doppler laser vibrometer (Model OFV-5000/534, Polytec, Germany) and a precision low-noise lock-in amplifier (MFLI5M, Zurich Instruments, Switzerland) under typical room temperature conditions. To achieve AFCs, the microcantilever resonator was resonantly stimulated by a sinusoidal AC pump at a frequency *f*_pump_ close to its primary flexural mode and a voltage *V*_pump_. The frequency response curves were collected by using a subtle sinusoidal AC probe signal at a frequency *f*_probe_ and a voltage *V*_probe_.

### Simulations

The air damping simulation was conducted under the air surrounded vibratory cantilever model (Fig. 2d) with FEM. The model contains a shell-cantilever beam placed in thermoviscous air, which are surrounded by pressure-acoustic perfectly matched layers (PML) for truncated calculations. A critical parameter in this model is the penetration depth *d* which represents the thickness of the viscous and thermal boundary layers. In the air, the two layers approximately share the same dimensions. Energy dissipation within the layers occurs via viscous drag and thermal conduction. The penetration depth *d* can be expressed as follows^41^: $d={\left(\mu /{\pi f}_{{\rm{r}}{\rm{\rho }}0}\right)}^{1/2}$, where $\mu$ is the air viscosity. The air damping loss is determined by using characteristic frequency analysis with the formula $2{\rm{Im}}\left(2\pi {f}_{{\rm{r}}}\right)/\mathrm{Re}({2\pi f}_{{\rm{r}}})$^42^.

## Supplementary information

Supplementary Information for Acoustic Frequency Comb Generation on a Composite Diamond/Silicon Microcantilever in Ambient Air

## Data Availability

The corresponding author’s data supporting this study’s findings are available upon reasonable request.
