# Supplementary material for: Acoustic frequency comb generation on a composite diamond/silicon microcantilever in ambient air
Source: Microsyst Nanoeng. 2025 Jan 17;11:12. doi: 10.1038/s41378-025-00866-x (PMC11739415; doi:10.1038/s41378-025-00866-x)
Supplement: Supplementary file 1 — Supplementary Information for Acoustic Frequency Comb Generation on a Composite Diamond/Silicon Microcantilever in Ambient Air [file 41378_2025_866_MOESM1_ESM.docx]

**Supplementary Information**

**Acoustic Frequency Comb Generation on a Composite Diamond/Silicon Microcantilever in Ambient Air**

Zhixin Zhao, Yanyan Li, Wangyang Zhang, Wenyao Luo, Duo Liu*

Institute of Novel Semiconductors, State Key Laboratory of Crystal Materials, Shandong University, 27 South Shanda Road, Shandong 250100, P. R. China

*Corresponding author: [liuduo@sdu.edu.cn](mailto:liuduo@sdu.edu.cn)

## Supplementary Note 1. Material characterization of diamond/silicon microcantilever beam.


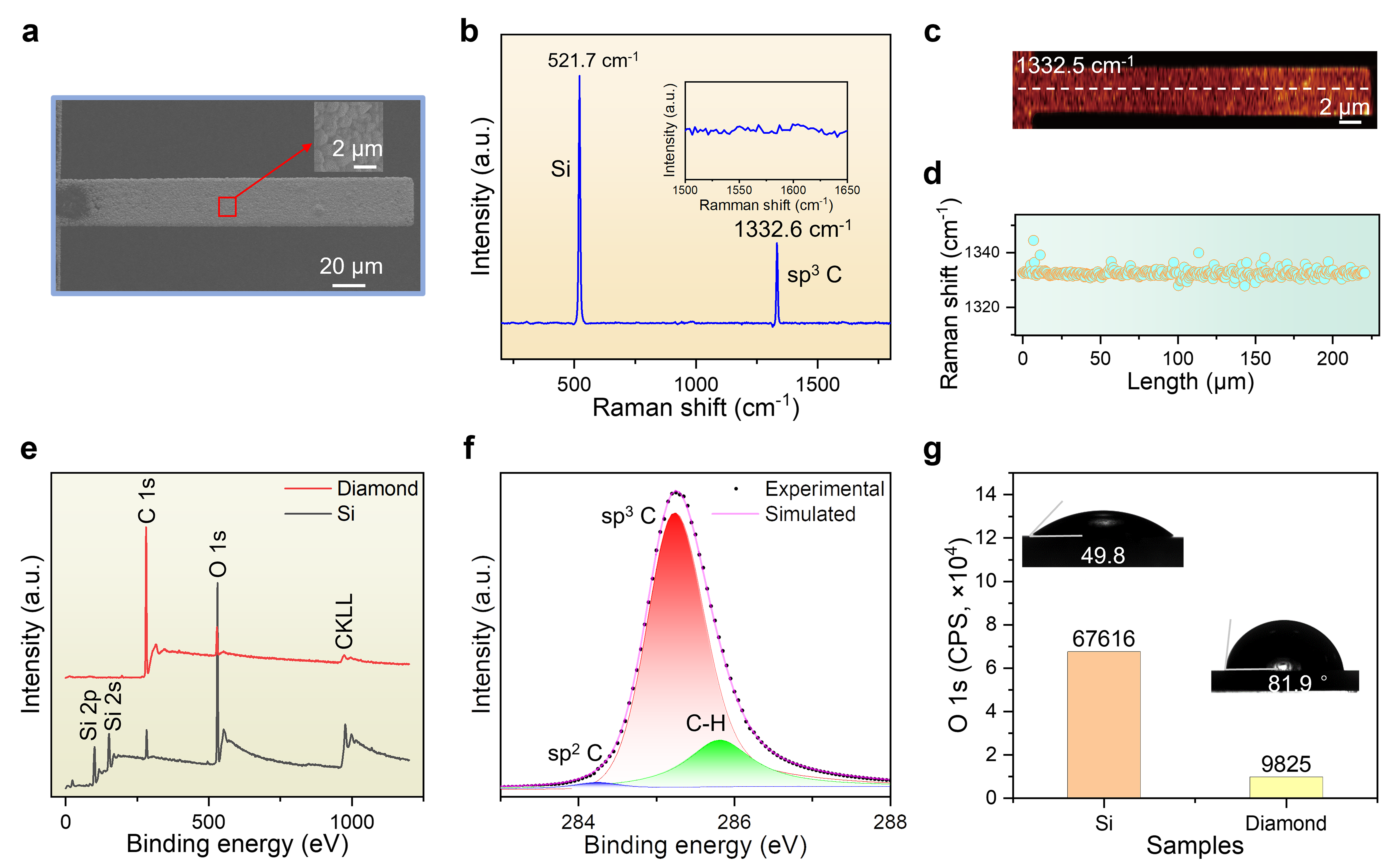


**Supplementary Fig. 1 Material characterization of** **diamond/silicon microcantilever beam. a** SEM image of the diamond/silicon microcantilever beam. The inset shows a partially enlarged view of the cantilever beam. **b** Raman spectrum of the microcantilever beam. **c** Raman intensity mapping image of the microcantilever at 1332 cm^-1^. **d** Raman peak position line profile obtained along the length of the microcantilever. The data were extracted from the white dashed line in panel (**c**). **e** XPS survey spectra obtained on the composite diamond/silicon film and the base of a silicon microcantilever. **f** C1s XPS spectra and fittings of diamond. **g** O1s CPS of silicon and diamond. The inset shows the contact angles of silicon and diamond respectively.

Supplementary Fig. 1a shows the SEM image of the diamond/silicon microcantilever beam, along with an enlarged view of the beam surface in the inset. The microcantilever surface remains flat and uniform after diamond deposition. Raman spectroscopy, known to have an exceptional sensitivity to the sp^2^ hybridized bonds, was utilized to

qualitatively assess the bonding properties of the diamond film^^[[1]](#endnote-1)^^. Supplementary Fig. 1b shows that the characteristic Raman peak for the diamond film is located at 1332.6 cm^-1^, which closely aligns with the distinctive Raman peak for unstressed single crystal diamond (1332 cm^-1^). The subtle blueshift of the Raman signals suggests existence of residual compressive stresses in the diamond layer. The lack of graphite peak at 1580 cm^-1^ signifies high crystallinity of the diamond layer ^^[[2]](#endnote-2)^^. As the Raman exaction laser (532 nm) can shine through the diamond layer, the Raman peak for silicon at 520 cm^-1^ was also detected. Supplementary Fig. 1c presents a Raman intensity mapping at 1332.5 cm^-1^, acquired on the microcantilever over an area measuring approximately 250 × 70 μm^2^, offering valuable insights into the spatial variations of the stress distribution on the microcantilever surface. Supplementary Fig. 1d depicts a Raman peak line profile extracted from the white dashed line in Supplementary Fig. 1c. The peak positions predominantly cluster around 1332.5 cm^-1^, suggesting a consistent stress distribution across the analyzed area. This observation implies that the material properties or stress levels within the microcantilever surface are relatively homogeneous.

The surface chemical composition and characteristics of both the diamond/silicon microcantilever and a silicon microcantilever were thoroughly examined through the utilization of XPS, which allowed for a detailed investigation into the elemental composition, chemical bonding, and surface chemistry of the microcantilevers, providing valuable insights into their surface properties and potential interactions with the surrounding environment. As shown in Supplementary Fig. 1e, the diamond layer on the diamond/silicon microcantilever contains only carbon (CKLL, C1s) and oxygen (O1s)^^[[3]](#endnote-3)^^, while the silicon microcantilever surface contains mainly silicon (Si2p, Si2s), oxygen (O1s) and carbon (CKLL, C1s). Interestingly, a notable distinction was observed in the oxygen content between the diamond/silicon microcantilever and the silicon microcantilever, with the former displaying a markedly lower oxygen content compared to the latter. This discrepancy in oxygen levels between the two microcantilevers highlights a distinct difference in their surface compositions and potentially indicates varying degrees of oxidation or surface contamination present on the microcantilever surfaces.

Supplementary Fig. 1f presents a high-resolution XPS spectrum of the C1s peak for the silicon microcantilever. The peak contains three sub-peaks located at 285.25, 284.20, and 285.80 eV, corresponding to sp^3^-C, sp^2^-C, and C-H chemical bonds^^[[4]](#endnote-4)^,^[[5]](#endnote-5)^^, respectively. The presence of hydrogen terminated carbon is typical for MPCVD diamond due to employment of hydrogen plasma during the growth process. The relatively low quantity of sp^2^-C content observed in the high-resolution XPS spectrum aligns well with the findings from the Raman spectrum, which can be attributed to the use of thermally refined NDs as the seeding layer and the subsequent hydrogen plasma treatment employed during the growth process. Both methods likely contribute to the suppression of sp^2^-C bonding, resulting in a predominantly sp^3^-C rich surface composition.

Supplementary Fig. 1g presents a compassion of the intensity of the oxygen O1s peaks extracted from Supplementary Fig. 1e for the two microcantilevers. The oxygen content on the diamond layer is approximately 6.9-times less than that of the silicon microcantilever due to the exceptional chemical inertness of diamond against oxidation in ambient air. Oxygen contents are usually hydrophilic in nature due to their tendency to form hydrogen bonds with water molecules. This is varied by water contact angle measurement conducted on a MPCVD diamond film and a silicon wafer. The insets of Supplementary Fig. 1g show the sideview images of a deionized water droplet (2.5 μL) on the diamond film and the silicon wafer, indicating that the diamond surface is more hydrophobic than the silicon surface. As we will discuss later, oxygen termination plays an essential role on surface loss during cantilever oscillation in ambient air, resulting from the adsorption and desorption of polar molecules.

## Supplementary Note 2. The calculation equation of each dissipation.

The analytic models used for the calculation have been developed for decades and ${\text{ }\text{Q}}_{\text{air}}^{\text{-1}}$^[[6]](#endnote-6)^, $\text{Q}_{\text{clamp }}^{\text{-1}}$^[[7]](#endnote-7)^, $\text{Q}_{\text{TED }}^{\text{-1}}$ ^[[8]](#endnote-8)^and $\text{Q}_{\text{surface }}^{\text{-1}}$^[[9]](#endnote-9)^ take the forms:

| $\text{Q}_{\text{air}}^{\text{-1}}\text{=}\frac{\text{3π}\text{μ}\left( \text{4 + }\text{w}_{\text{c}}\sqrt{{\text{2}\text{ρ}}_{\text{0}}\text{ω}\text{/}\text{μ}} \right)}{\text{4}\text{ρ}_{\text{c}}\text{l}_{\text{c}}\text{t}_{\text{c}}\text{ω}}\text{, }\text{ω}\text{=3.516}\frac{\text{t}_{\text{c}}}{\text{l}_{\text{c}}^{\text{2}}}\sqrt{\frac{\text{E}_{\text{c}}}{\text{12}\text{ρ}_{\text{c}}}}$ | (S1) |
| --- | --- |
| $\text{Q}_{\text{clamp }}^{\text{-1}}\text{=}\frac{\text{β}\text{w}_{\text{c}}}{\text{l}_{\text{c}}}\left( \frac{\text{t}_{\text{c}}}{\text{t}_{\text{b}}} \right)^{\text{2}}$ | (S2) |
| $\text{Q}_{\text{TED }}^{\text{-1}}\text{=}\frac{\text{α}_{\text{T}}^{\text{2}}\text{T}\text{E}_{\text{c}}}{\text{C}_{\text{p}}}\frac{\text{ω}\text{τ}_{\text{R}}}{\text{1 +}\text{ ω}^{\text{2}}\text{τ}_{\text{R}}^{\text{2}}}\text{, }\text{τ}_{\text{R}}\text{=}\frac{\text{t}_{\text{c}}^{\text{2}}\text{ρ}_{\text{c}}\text{C}_{\text{p}}}{\text{π}^{\text{2}}\text{κ}}$ | (S3) |
| $\text{Q}_{\text{surface }}^{\text{-1}}=\frac{2\left( 3w_{c} \text{+} t_{c} \right)}{w_{c}t_{c}}\frac{\chi\text{E}^{\text{S}}}{E_{c}}$ | (S4) |

where $\text{w}_{\text{c}}$*,* $\text{t}_{\text{c}}$*,* $\text{l}_{\text{c}}$*,* $\text{ ρ}_{\text{c}}$*,* $\text{E}_{\text{c}}$and $\text{ω}$ are the width, thickness, length, density, the Young’s modulus and vibrational frequency of the cantilevered beam, respectively; $\text{ρ}_{\text{0}}$ is the air density, $\text{μ}$ is the air viscosity; $\text{β}$ is related to Poisson’s ratio and flexural wavenumber of the cantilever, which approximately equals to 0.95, $\text{t}_{\text{b}}$ is thickness of the supported base; $\text{C}_{\text{p}}$ is the heat capacity per unit volume at constant pressure, $\text{α}_{\text{T}}$ is the thermal expansion coefficient, $\text{κ}$ is the thermal conductivity, and *T* is the temperature at which the microcantilever operating; $\text{E}^{\text{S}}$ and $\text{χ}$ are the dissipative Young’s modulus and the thickness of the surface layer, respectively.

Note that, for the calculation of the surface loss for the silicon microcantilever, the microcantilever is assumed to be covered by a layer of silica with a dissipative Young’s modulus of 2 GPa^^[[10]](#endnote-10)^^, and a thickness of 2 nm^^[[11]](#endnote-11)^^, which resulting in a $\text{χ}\text{E}^{\text{S}}$ of 4 Pa·m. As a result, the calculation accounts only for the surface loss from a mechanically deformed surface layer^^[[12]](#endnote-12)^^ and does not include surface losses due to surface adsorption of polar molecules in the air^^[[13]](#endnote-13)^^. It is expected that the actual surface loss would be greater than the calculated values shown in Fig. 2c.

The parameters used for the calculations are listed in Supplementary Table 1.

**Supplementary Table 1.** The parameters used in the calculation of the *Q* factors shown in Fig. 2c.

| $\text{μ}$  (10^-5^Pa·s) | $\text{ρ}_{\text{0}}$ (kg/m³) | $\text{t}_{\text{b}}$ (mm) | $\text{α}_{\text{T}}$  (10^-6^/K) | $\text{C}_{\text{p}}$  (J/(kg·K)) | $\text{κ}$  (W/(m·K)) | $\text{χ}\text{E}^{\text{S}}$  (Pa·m) | $\text{E}_{\text{c}}$ (GPa) |
| --- | --- | --- | --- | --- | --- | --- | --- |
| 1.81 | 1.21 | 0.6 | 2.6 | 700 | 130 | 4 | 170 |

## Supplementary Note 3. The dynamics of the system in a self-sustained resonator.

The dynamics of the system in a self-sustained resonator with forced vibration is given^[[14]](#endnote-14)^：

| $\dot{\text{A}}\text{=}\frac{\text{λ}}{\text{2}}\text{A}\text{-}\frac{\text{1}}{\text{8}}\text{A}^{\text{3}}\text{-}\frac{\text{B}}{\text{2}\text{ω}_{\text{pump}}}\sin\text{δφ}\text{,}$ | (S5) |
| --- | --- |
| $\dot{\text{δφ}}\text{=}\text{∆}\text{-}\frac{\text{B}}{\text{2}\text{A}\text{ω}_{\text{pump}}}\cos\text{δφ}.$ | (S6) |

where $\text{A}$ represents the vibration amplitude, $\text{λ}$ the damping coefficient, $\text{B}$ the amplitude of the external forcing, and $\text{∆}\text{≈(}\text{ω}_{\text{r}}\text{-}\text{ω}_{\text{pump}}\text{)}$ and $\text{δφ}$ the frequency and phase difference between the model frequency and the periodic pumping frequency, respectively.

## Supplementary Note 4. Sample fabrication and experimental setup.


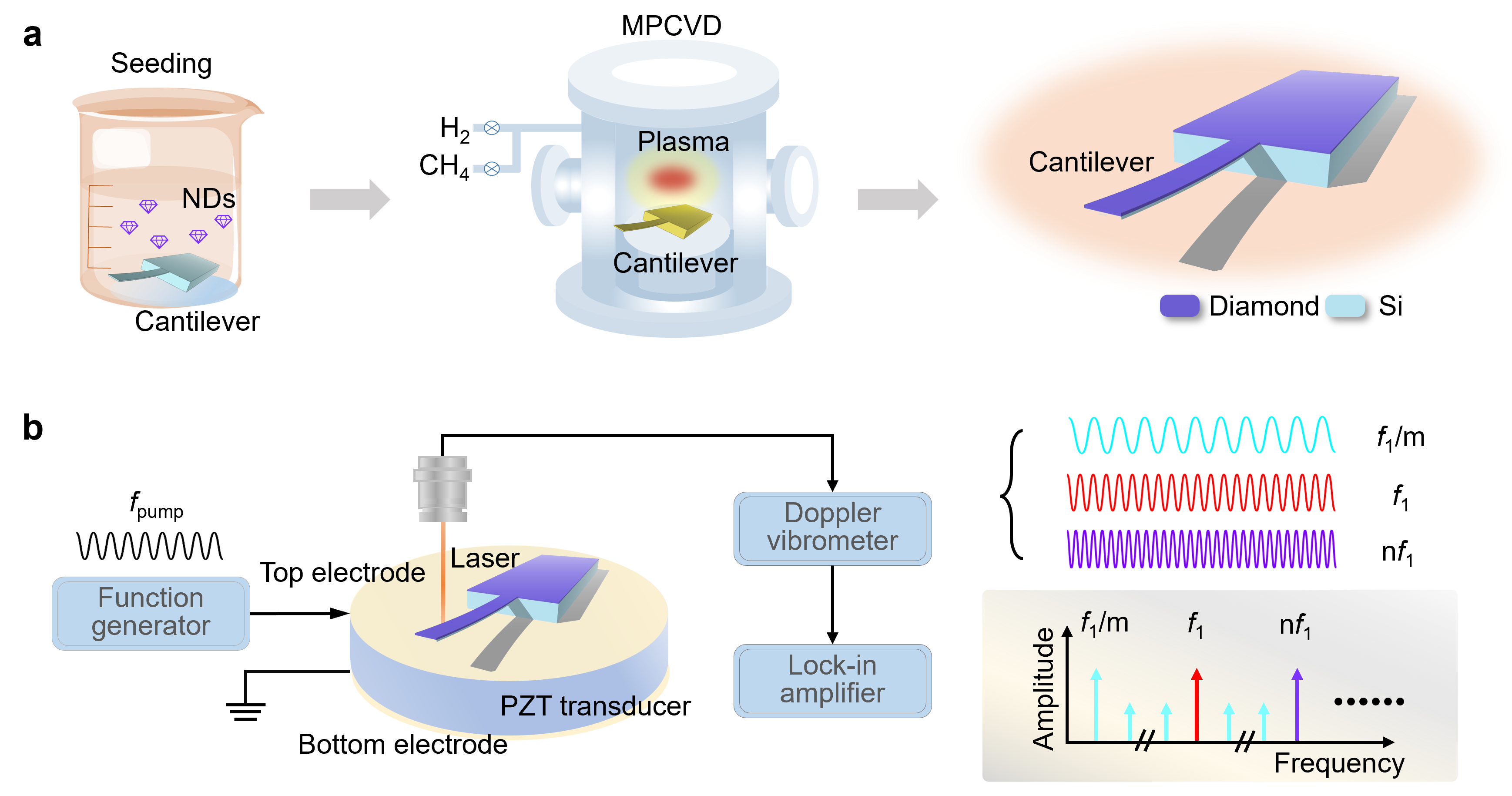


**Supplementary Fig. 2. Sample fabrication and experimental setup. a** Schematic of the experimental steps used to depositing thin diamond layer on silicon microcantilever. **b** Schematic of the experimental setup.

This relevant explanation can be found in the 'Materials and Methods' section of the main text.

## References

1. . Campion, A. Vibrational Spectroscopy of Molecules on Surfaces (Springer-Verlag, 1987). [↑](#endnote-ref-1)
2. . Stuart, S. A., Prawer, S. & Weiser, P. S. Growth‐sector dependence of fine structure in the first‐order Raman diamond line from large isolated chemical‐vapor‐deposited diamond crystals. *Appl. Phys. Let.* **62**, 1227-1229 (1993). [↑](#endnote-ref-2)
3. . Thomas, E. L. H., Nelson, G. W., Mandal, S., Foord, J. S. & Williams, O. A. Chemical mechanical polishing of thin film diamond. *Carbon* **68**, 473-479 (2014). [↑](#endnote-ref-3)
4. . Ferro, S., Dal Colle, M. & De Battisti, A. Chemical surface characterization of electrochemically and thermally oxidized boron-doped diamond film electrodes. *Carbon* **43**, 1191-1203 (2005). [↑](#endnote-ref-4)
5. . Graupner, R., Maier, F., Ristein, J., Ley, L. & Jung, C. High-resolution surface-sensitive C1s core-level spectra of clean and hydrogen-terminated diamond (100) and (111) surfaces. *Phys. Rev. B* **57**, 12397-12409 (1998). [↑](#endnote-ref-5)
6. . Blom, F. R., Bouwstra, S., Elwenspoek, M. & Fluitman, J. H. J. Dependence of the quality factor of micromachined silicon beam resonators on pressure and geometry. *J. Vac. Sci. Technol., B: Microelectron. Nanometer Struct. Process., Meas., Phenom.* **10**, 19-26 (1992). [↑](#endnote-ref-6)
7. . Photiadis, D. & Judge, J. Attachment losses of high Q oscillators. *Appl. Phys. Let.* **85**, 482-484 (2004). [↑](#endnote-ref-7)
8. . Zener, C. Internal friction in solids. I. Theory of internal friction in reeds. *Phys. Rev.* **52**, 230-235 (1937). [↑](#endnote-ref-8)
9. . Yasumura, K. Y. *et al.* Quality factors in micron- and submicron-thick cantilevers. *J. Microelectromech. Syst.* **9**, 117-125 (2000). [↑](#endnote-ref-9)
10. . Klumbach, S. & Schilling, F. R. Anisotropic viscoelastic properties of quartz and quartzite in the vicinity of the α–β phase transition. *Phys. Chem. Miner.* **44**, 627-637 (2017). [↑](#endnote-ref-10)
11. . Morita, M., Ohmi, T., Hasegawa, E., Kawakami, M. & Ohwada, M. Growth of native oxide on a silicon surface. *J. Appl. Phys.* **68**, 1272-1281 (1990). [↑](#endnote-ref-11)
12. . Ibach, H. The role of surface stress in reconstruction, epitaxial growth and stabilization of mesoscopic structures. *Surf. Sci. Rep.* **29**, 195-263 (1997). [↑](#endnote-ref-12)
13. . Ibach, H. Adsorbate‐induced surface stress. *J. Vac. Sci. Technol., A* **12**, 2240-2245 (1994). [↑](#endnote-ref-13)
14. . Balanov, A. G., Janson, N. B., Postnov, D. E. & Sosnovtseva, O. V. *Synchronization: from simple to complex* (Springer-Verlag, 2008). [↑](#endnote-ref-14)
